# Micronutrient inadequacy among lactating mothers in rural areas of North Mecha District, Amhara Region, Ethiopia

**DOI:** 10.3389/fnut.2024.1354459

**Published:** 2024-03-20

**Authors:** Yonatan Menber, Selamawit Gashaw, Tefera Belachew, Netsanet Fentahun

**Affiliations:** ^1^Department of Nutrition and Dietetics, School of Public Health, College of Medicine and Health Sciences, Bahir Dar University, Bahir Dar, Ethiopia; ^2^Department of Nutrition and Dietetics, Faculty of Public Health, College of Public Health, Jimma University, Jimma, Ethiopia

**Keywords:** dietary intake, nutrient inadequacy, lactating mothers, North Mecha, Amhara, Northwest Ethiopia

## Abstract

**Background:**

Lactating mothers are frequently at risk for nutritional deficiencies due to the physiological requirements of lactation. Throughout the world, a significant number of lactating mothers have micronutrient intake inadequacy. Evidence on micronutrient intake during lactation is limited in rural Ethiopia. Therefore, this study aimed to determine micronutrient intake inadequacy and associated factors among lactating mothers.

**Methods and materials:**

A community-based cross-sectional study was conducted from February 1 to 18, 2023, among lactating mothers in rural areas of the North Mecha District of Amhara Region, Northwest Ethiopia. A multistage sampling technique was used to select 449 study participants. An interviewer-administered questionnaire was employed to collect dietary intake data by using a single multiphasic interactive 24-h dietary recall. The NutriSurvey 2007 software and Ethiopia, Tanzania and Kenya food composition tables were used to calculate nutrient values for the selected 12 micronutrients. For the remainder of the analysis, SPSS version 25 was employed. The Nutrient Adequacy Ratio (NAR) and Mean Adequacy Ratio (MAR) were calculated by dividing all NAR values by the number of micronutrients computed to evaluate the nutrient intakes. A logistic regression analysis was conducted to determine the factors contributing to the overall micronutrient intake inadequacy, and statistical significance was determined at a *p* value <0.05.

**Result:**

A total of 430 lactating mothers participated in the study, with a 96% response rate and a mean age of 29.46 ± 5.55 years. The overall prevalence of micronutrient intake inadequacy was 72.3% (95% CI: 67.9, 76.5). The odds of micronutrient intake inadequacy were 2.5 times higher among lactating mothers aged 18–25 years old as compared to mothers in the age group ≥36 years old (AOR = 2.52, 95% CI: 1.09, 5.83). Mothers with the educational status of unable to read and write and primary school incomplete were 3.5 (AOR = 3.49, 95% CI: 1.24, 9.83) and 3.6 (AOR = 3.56, 95% CI: 1.06, 11.99) times more likely to have micronutrient intake inadequacy than mothers with secondary school completed or above educational status, respectively. Mothers whose partner’s occupation was other than farming were 3.3 times more likely to have micronutrient intake inadequacy as compared to mothers whose partners were engaged in farming (AOR = 3.32, 95% CI: 1.08, 10.27). Lactating mothers who were from food-insecure households were 83% more likely to have high micronutrient intake inadequacy as compared to lactating mothers from food-secure households (AOR = 1.83, 95% CI: 1.04, 3.23). Lactating mothers with nutrition-related unfavorable attitudes were 77% more likely to have inadequate intake of micronutrients compared to lactating mothers with favorable attitudes (AOR = 1.77, 95% CI: 1.07, 2.93).

**Conclusion:**

The prevalence of micronutrient intake inadequacy among lactating mothers was high. Age of the mothers, educational status of the mothers, occupation of the partner, household food security, and nutrition-related attitude were significantly associated with micronutrient intake inadequacy. Community driven nutrition education and interventions are needed to address the high micronutrient intake inadequacy among lactating mothers in rural Ethiopia.

## Introduction

The foundation of a healthy human diet is comprised of macronutrients that serve as the body’s primary energy source and micronutrients that are required for nearly all metabolic and developmental processes (1). Nutritional adequacy is defined as the sufficient intake of essential macronutrients and micronutrients needed to fulfill nutritional requirements for optimal health (2).

As nutritional needs alter over the course of life and physiological status, the recommended intake of micronutrients for mothers is comparatively higher during lactation (3). It is crucial for mothers to consume a varied and balanced diet throughout this crucial age window. The nutritional quality and adequacy of the mother’s diet have a major impact on the amount and content of breast milk produced. Variations in the mother’s diet can result in alterations in the levels of specific micronutrients. However, in the case of certain other nutrients, the mother’s body prioritizes the baby’s requirements, resulting in the secretion of these specific nutrients at their highest potential, at the expense of maternal stores. This signifies that inadequate intake of micronutrients can have a negative impact on a mother’s present health and future susceptibility to disease, as well as a child’s growth, development, and overall health, including later in life. As a result, a breastfeeding woman needs a diet packed with nutrients (4–7).

The majority of lactating mothers had inadequacy for many of the micronutrients, with a global prevalence of micronutrient inadequacy reaching over 50%. Most lactating mothers (87.2–97.6%) in rural Bangladesh did not meet the recommended levels of riboflavin, calcium, vitamin A, and folate (8). Lactating mothers in Brazil had a high prevalence of micronutrient inadequacy, even more so than mothers of reproductive age, for vitamin A (95% versus 72%), vitamin C (56% versus 37%), vitamin B6 (75% versus 33%), folate (72% versus 40%), and zinc (64% versus 20%) (9). Mothers were at risk of consuming insufficient amounts of folate and vitamins A, D, and E, with 87.0, 93.4, 43.8, and 95% of mothers consuming less than the Estimated Average Requirement (EAR) for these nutrients among Hispanic lactating mothers in Southern California (10). Studies carried out in Africa revealed that more than half of lactating mothers were susceptible to consuming inadequate amounts of micronutrients (11, 12).

A study conducted among lactating mothers in the Genta Afeshum District of the Tigray Region, North Ethiopia, revealed that a significant proportion of the mothers were affected by micronutrient inadequacy. The inadequacies observed were as follows: vitamin A (79.5%), B1 (28.4%), B3 (58.8%), B6 (95.5%), B12 (99.9%), C (83.9%), calcium (99.9%), iron (24.6%), and zinc (58.3%) (13). Another study carried out among lactating mothers in Bahir Dar city of the Amhara Regional State, Northwest Ethiopia, also highlighted high levels of micronutrient inadequacy among mothers in the city. The study found that the following percentages of city mothers had inadequate levels of the respective micronutrients: vitamin A (98.2%), B1 (13.4%), B2 (54.3%), B3 (42.3%), B6 (65.6%), B9 (25.2%), B12 (68.5%), C (73.8%), calcium (70.9%), iron (0.0%), zinc (4.7%), and selenium (36%) (14).

Nutrition programs have been launched worldwide with the goal of decreasing diverse forms of malnutrition. By 2030, the Sustainable Development Goal (SDG2) aims to reduce malnutrition in all its forms (15). The World Health Organization (WHO) has set global targets for improving the nutrition of mothers, infants, and young children, and member states have committed to tracking these targets, as stated in Global Targets 2025 (16). The Ethiopian government had exerted many efforts with the goal of decreasing diverse forms of malnutrition, including conducting an Ethiopian national micronutrient survey (17). The government of Ethiopia has also developed the Food and Nutrition Policy (FNP) (18). The Seqota Declaration reflects the commitment to ending undernutrition by 2030 (19, 20). Despite the various forms of interventions implemented by the government of Ethiopia, malnutrition reduction is steady, and millions remain malnourished (21).

This study focuses on lactating mothers who have increased nutritional demands due to their breast milk production. This makes them highly vulnerable to malnutrition, thus making it crucial to ensure the health of both the mother and the infant. Likewise, micronutrient inadequacy is a significant public health concern among rural lactating mothers. There is a paucity of evidence on the micronutrient inadequacy of lactating mothers in rural Ethiopia. Therefore, this study aimed to determine micronutrient inadequacy and its factors among lactating mothers in rural areas of the North Mecha District of the Amhara Region, Northwest Ethiopia.

## Materials and methods

### Study setting and study design

The study was conducted in North Mecha District, North Gojam Zone, Amhara Regional State, Northwest Ethiopia. The district is located 530 km from Addis Ababa, the capital city of Ethiopia. Predominantly, North Mecha District is dependent on agriculture, which is a livelihood for 85% of the total population. The district is well-known for its crop production, including teff, maize, barely, wheat, beans, and peas as major crops using rainfall as well as irrigation. There is also production of fruits and vegetables (22). Koga, one of the large-scale dams with 7,000 hectar irrigation capacity, is found in the district. The Koga Irrigation and Watershed Management Project is a government initiative designed in the district to increase agricultural productivity and improve water management in the Koga watershed area of Ethiopia, with a focus on poverty reduction and food security. The project is expected to have a positive impact on food consumption and nutrition practices in the region, thereby promoting human and economic development (23). A community-based cross-sectional study design was employed from February 1 to 18, 2023.

### Population and eligibility criteria

Lactating mothers who had children of 6–23 months of age and who were living in North Mecha District were the source population, and lactating mothers who were living in the selected kebeles of the district were the study population. Lactating mothers who had been residents of the study area for at least 6 months were included in the study, and mothers who were currently pregnant were excluded from the study. Mothers who had fasted or participated in special events such as festivals or times of mourning in the last 24 h were excluded from the study.

### Sample size and sampling technique

A single population proportion formula was used to determine the sample size with the following assumptions: a 95% confidence level, a 5% margin of error, and a 77% proportion of inadequate dietary intake among lactating mothers from a prior study carried out in the Finote Selam District, Northwest Ethiopia (24). After multiplying by a design effect of 1.5 and including a 10% non-response rate, the final sample size was 449. A multistage sampling technique was used to select the study participants. Out of a total of 38 kebeles, seven were randomly selected through a lottery system. Following the proportional allocation of lactating mothers to each kebele, which was determined by taking into account the total number of lactating mothers residing in each selected kebele and across all selected kebeles, study participants were chosen from the selected kebeles using a systematic random sampling approach. The calculation of the parameter ‘K’ was conducted by dividing the study population (N) by the desired sample size (n).

### Operational definitions

Recommended Dietary Allowances (RDA): This is the daily intake that meets the nutrient requirements of almost all (97.5 percent) lactating mothers (25).

Nutrient Adequacy Ratio (NAR): It is the ratio of a subject’s intake of a micronutrient to the current recommended daily allowance for each sex and age category (26).

Mean Adequacy Ratio (MAR): It is an indicator of the overall quality of a diet. It is calculated by dividing all NAR values by the number of micronutrients computed (26).

Micronutrient intake inadequacy: It is when a lactating mother’s intake of NAR for a particular micronutrient is less than 1 (100%) (26).

Overall micronutrient intake inadequacy: The ideal MAR cut-off for nutrient intake adequacy should be one (100%), which would mean that the intake of all 12 nutrients, namely vitamin A, vitamin B1, vitamin B2, vitamin B3, vitamin B6, vitamin B9, and vitamin B12, vitamin C, calcium, iron, zinc, and selenium, is equal to or greater than the RDA and the requirements for all the nutrients are met. Iron was adequate for all study participants, and the final calculation of MAR was carried out by excluding the universally adequate nutrient, iron. Consequently, the prevalence of micronutrient intake was computed among the remaining 11 nutrients after excluding iron. In this study, since there was no participant who had a MAR score of 1, overall micronutrient intake inadequacy was operationalized to be <0.75 (26–28).

Household Food Insecurity Access Scale (HFIAS) score: It is calculated for each household as the sum of the frequency of occurrences during the past 4 weeks for the nine-food insecurity-related conditions. It can be scored and classified as food secure, mildly food insecure, moderately food insecure, and severely food insecure (29).

Wealth Index: The wealth index of households was determined using the Principal Component Analysis (PCA) by considering various household assets, housing conditions, access to services, and other variables adapted from the 2019 Ethiopian Mini Demographic and Health Survey (21). The collected data on these variables were transformed into binary (yes/no) variables and coded as 1 for ‘yes’ and 0 for ‘no.’ To ensure the accuracy of the wealth index score, the assumptions of PCA were thoroughly examined. The eigenvalue-one criterion was employed to determine the number of components to retain. Furthermore, only variables with a commonality value exceeding 0.5 were utilized to generate factor scores. To create the wealth score, the score for each household on the first principal component was retained. Finally, quintiles were applied to the wealth score in order to classify households into categories such as poorest, poor, medium, rich, and richest (30).

Nutrition-related knowledge: It was determined using a questionnaire developed in accordance with FAO guidelines for assessing nutrition-related knowledge. To generate scores, each question was given equal weight, with a score of either 1 or 0 assigned to it. A correct response received a score of 1, while an incorrect answer to the question results in a score of 0. The final score, indicating the level of nutrition-related knowledge, was calculated by considering the percentage of correct responses out of the total number of questions used for assessment. A score of ≥75% was considered adequate knowledge, and < 75% indicated inadequate knowledge (31–34).

Nutrition-related attitude: It was determined using a questionnaire developed in accordance with FAO guidelines for assessing nutrition-related attitude. To generate scores, each question was given equal weight, with a score of either 1 or 0 assigned to it. Among the three-point Likert scale questions for the attitude assessment, the first positive response was given a score of 1, while the second and third responses were assigned a score of 0. The final score, indicating the level of nutrition-related attitude, was calculated by considering the percentage of favorable responses out of the total number of questions used for assessment. A score ≥ 75% was considered favorable attitude, and < 75% indicated unfavorable knowledge (31–34).

### Data collection tools and procedure

An interviewer-administered semi-structured questionnaire was used to collect data on socio-demographic and economic factors; water, sanitation, and hygiene; household food security; knowledge and attitude of mothers on nutrition; and health-related factors. The data were gathered using the Kobo Tool Box, an electronic data collection toolkit. The dietary data were assessed using the Food and Agriculture Organization of the United Nations (FAO) standardized tool, the 24-h recall dietary data collection tool (35). Nutrition-related knowledge and attitude data were collected using a questionnaire developed in accordance with FAO guidelines for assessing nutrition-related knowledge, attitudes, and practices (33). Ten data collectors and two supervisors who had a background in public health nutrition were involved in data collection and supervision.

### 24-h dietary recall assessment

Prior to actual data collection, inspections of the market and home surveillance were done to collect data on the types of foods eaten, cooking methods, and household utensils used in the study area. Photographs of household utensils and food portions usually eaten at one meal were taken during surveillance, and then codes were assigned to each. In the nutrition laboratory, utensils used for food serving were standardized with food portions and water using a digital food portion weighing scale and a measuring graduated cylinder.

During the actual data collection, the respondents were asked which utensil they used from the photographic atlas. Photographs depicting various types and sizes of household utensils (spoons, ladles, cups, glasses, plates, and bowls) and food portions were used to assist the participants in recalling and determining the types and portion sizes of the consumed items using an interactive multiple-pass 24-h recall method. The quantities of foods consumed were estimated using household utensils and numbers, such as the numbers of oranges, bananas, mangoes, potatoes, etc. Food items quantified in numbers were collected as large, medium, and small.

### Data quality control

The questionnaire was developed in English, translated into Amharic, and then back into English to ensure consistency. The data collection tool included standard questions developed by FAO as well as questions adapted from other sources and included in the tool after content validation by field experts. Based on their recommendations, any modifications that were required were made. A pretest was done on about 5% of the sample. Training was provided to supervisors and data collectors. The data collection process was followed with close supervision, and the completeness of the data was checked accordingly. To help the participants remember and identify the kinds and amounts of food they had consumed, photographs of food portions and household utensils (spoons, ladles, cups, glasses, etc.) were used.

A multiple-pass 24-h recall with three passes was carried out to collect dietary intake data. In the three sequential stages of multiple passes, a “quick list,” a “detailed description of food and beverage items consumed,” and a “review” were conducted.

### Data processing and analysis

Following the completion of the data collection period, the obtained food consumption data were converted into nutrient intake data. The Ethiopian food composition tables were used to get nutrient values per 100 grams of each food item (36, 37). The nutrient value for certain food items that are not included in Ethiopian food composition tables was supplied using food composition tables from other African countries (Kenya and Tanzania) (38, 39). When utilizing food composition tables from Kenya and Tanzania, certain assumptions were taken into account. The methods employed in generating the tables adhere to standardized protocols and ensure that the referred food items and portion sizes are comparable. SPSS version 25 was used for analysis, and the nutrient intake analysis was conducted, particularly using NutriSurvey 2007 software.

The RDA established by WHO and FAO in 2004 was compared to nutrient intakes, and the inadequacy of a particular nutrient was calculated using the Nutrient Adequacy Ratio (NAR), whereas the overall micronutrient intake inadequacy was measured using the Mean Adequacy Ratio (MAR) for 12 micronutrients, which are selected based on their public health importance, namely vitamin A, thiamin, riboflavin, niacin, vitamin B-6, folate, vitamin B-12, vitamin C, calcium, iron, zinc, and selenium (26, 40).


$$NAR=\frac{\mathrm{Actual}\;\mathrm{intake}\;\mathrm{of}\;\mathrm{the}\;\mathrm{nutrient}\;\mathrm{per}\;\mathrm{day}\;}{RDA\;\mathrm{of}\;\mathrm{that}\;\mathrm{nutrient}}$$

As an overall measure of micronutrient adequacy, the MAR was calculated as:


$$MAR=\frac{\sum NAR\;\left(\mathrm{each}\;\mathrm{truncated}\;\mathrm{at}\;1\right)}{\mathrm{Number}\;\mathrm{of}\;\mathrm{nutrients}}$$

NAR was truncated at 1, so that a nutrient with a high NAR could not compensate for a nutrient with a low NAR. For NAR, it was considered adequate if the intake of each nutrient was equal to or above the RDA. For MAR, it was considered adequate if the ratio of the sum of the NAR of each nutrient to the total number of nutrients was equal to 1 (26). Micronutrient intake adequacy data were checked by the Kolmogrov Smirnov and Shapiro Wilk tests of normality; the median and interquartile range were used to present the findings with a skewed distribution.

To identify the factors associated with lactating mothers’ overall micronutrient intake inadequacy, a binary logistic regression analysis was performed. To determine their independent effect, variables found in bivariable binary logistic regressions with a *p*-value ≤0.25 were entered into the multivariable binary logistic regression analysis. A *p*-value greater than 0.05 indicates a good fit in multivariable binary logistic regression models, which were tested for model fitness using the Hosmer-Lemeshow goodness of fit test (*p*-value = 0.867). In a multivariable binary logistic regression analysis, variables with a p-value of less than 0.05 were considered to be statistically significant. The Adjusted Odds Ratio (AOR) with a 95% Confidence Interval (CI) was used to express the degree of association between a dependent variable and independent variables. Texts, tables, and graphs were used to present the final results.

## Results

### Socio-demographic and socioeconomic characteristics

A total of 430 lactating mothers participated in the study, with a 96% response rate and a mean age of 29.46 ± 5.55 years. With regard to their educational status, 74.2% of participants were unable to read and write. Little over half (52.1%) had a family size of 5–7 people, and the mean family size was 5.81 ± 1.90. The majority were married (98.4%) and farmers (77.4%). The mean (± SD) of family size, parity, and number of children were 5.81 (± 1.90), 3.94 (± 2.01), and 1.31 (± 0.48), respectively (Table 1).

**Table 1:** Socio-demographic characteristics among lactating mothers in North Mecha District, Amhara Region, Ethiopia, 2023 (*N* = 430).

Variables	Categories	Frequency	Percentage
Maternal age (years)	18–25	119	27.7
	26–35	258	60.0
	≥36	53	12.3
Religion	Orthodox	430	100
Maternal education	Unable to read and write	319	74.2
	Primary school incomplete	40	9.3
	Primary school completed	42	9.8
	Secondary school completed	24	5.6
	University or college completed	5	1.2
Maternal occupation	Farmer	333	77.4
	Merchant	17	4.0
	Housewife	78	18.1
	Employee	2	0.5
Maternal marital status	Married	423	98.4
	Widowed	7	1.6
Partner education (423)	Unable to read and write	193	44.9
	Primary school incomplete	165	38.4
	Primary school completed	44	10.2
	Secondary school completed	17	4.0
	University or college completed	4	0.9
Partner occupation (423)	Farmer	390	90.7
	Merchant	15	3.5
	Student	4	0.9
	Daily laborer	7	1.6
	Other^#^	7	1.6
Family size	≤4	123	28.6
	5–7	224	52.1
	≥8	83	19.3
Parity	≤2	123	28.6
	3–5	209	48.6
	≥6	98	22.8
Number of <5 children	1	300	69.8
	≥2	130	30.2
Wealth Index	Poorest	86	20.0
	Poor	84	19.5
	Medium	85	19.8
	Rich	90	20.9
	Richest	85	19.8

^#^Driver, Soldier, Veterinarian.

### Nutrition-related factors

About 17.7 and 67.0% of lactating mothers had adequate knowledge and a favorable attitude, respectively. Three hundred twenty-one (74.7%) lactating mothers were from food-secured households (Table 2).

**Table 2:** Household food security, nutrition-related knowledge and attitudes among lactating mothers in North Mecha District, Amhara Region, Ethiopia, 2023 (*N* = 430).

Variables	Categories	Frequency	Percentage
Nutrition-related knowledge	Adequate	76	17.7
	Inadequate	354	82.3
Nutrition-related attitude	Favorable	288	67.0
	Unfavorable	142	33.0
Household food security	Food Secure	321	74.7
	Mildly food insecure	33	7.7
	Moderately food insecure	64	14.9
	Severely food insecure	12	2.8

### Micronutrient intake inadequacy among lactating mothers

**Figure 1:**
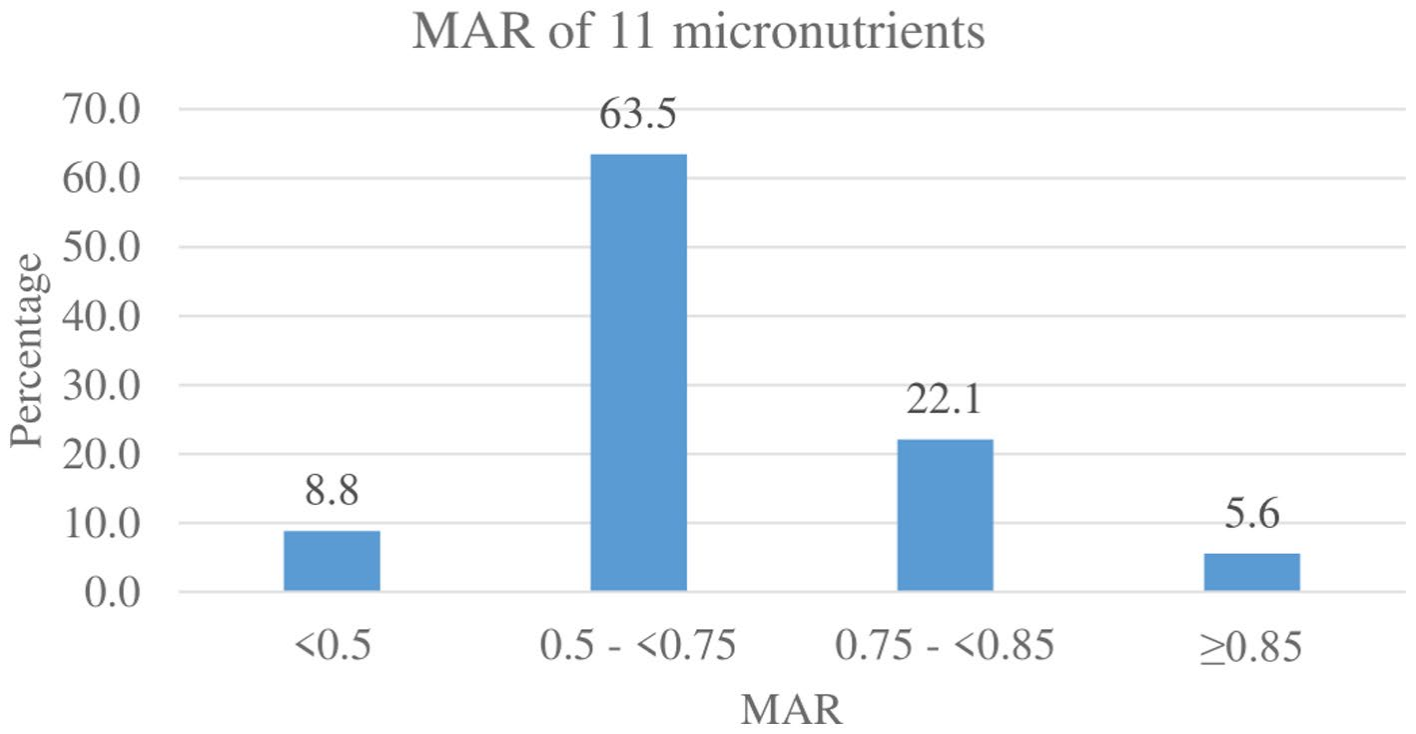
MAR of micronutrient intake among lactating mothers in North Mecha District, Amhara Region, Ethiopia, 2023 (*N* = 430).

Iron intake adequacy was 100% prevalent; all mothers had NAR greater than or equal to one. With the exception of iron, all micronutrient intakes by lactating mothers did not meet the recommended levels (MAR of one), resulting in MAR ranges from 0.29 to 0.94 for the rest of the 11 nutrients. The overall prevalence of micronutrient intake inadequacy, MAR < 0.75, was 72.3% (95% CI: 67.9, 76.5). This prevalence was computed among 11 nutrients after excluding iron, which was adequate for all participants (Figure 1).

The median intake of vitamin A among respondents was 292.9 μg (IQR: 326.5), with an inadequacy prevalence of only 86.7% (95% CI: 83.5, 90.0). The median intake of vitamin B9, vitamin C, and iron was 365.3 mcg (IQR: 645.2), 14.4 mg (IQR: 14.2), and 272.7 mg (IQR: 125.5), respectively. The proportion of lactating mothers with a nutrient intake below the RDA varied between the nutrients. None of the participants had iron intake inadequacy. In contrast, Ca intake inadequacy prevalence was 98.4% (95% CI: 97.0, 99.5), which was the maximum as compared to all other nutrients (Table 3).

**Table 3:** Micronutrient intake among lactating mothers in North Mecha District, Amhara Region, Ethiopia, 2023 (*N* = 430).

Micronutrient	RDA	Actual intake		NAR		Prevalence of micronutrient inadequacy (%) and (95% CI)
		Median	IQR (Q3-Q1)	Median	IQR (Q3-Q1)	
Vitamin A(μg)	850	292.9	326.5	0.34	0.38	86.7(83.5,90.0)
Vitamin B1(mg)	1.5	3.4	5.0	2.27	3.33	33.7(29.3,38.4)
Vitamin B2(mg)	1.6	1.4	1.2	0.88	0.75	57.4(52.8,62.1)
Vitamin B3(mg)	17	10.7	7.4	0.63	0.44	80.5(76.5,84.0)
Vitamin B6(mg)	2	2.8	1.5	1.40	0.75	18.1(14.7,22.1)
Vitamin B9 (mcg)	500	365.3	645.2	0.73	1.29	60.7(56.3,65.6)
Vitamin B12 (mcg)	2.8	<0.001	0.9	<0.001	0.32	82.3(78.6,85.6)
Vitamin C(mg)	70	14.4	14.2	0.21	0.20	98.4(97.0,99.5)
Calcium(mg)	1,000	614.6	314.9	0.61	0.31	92.8(90.0,95.1)
Iron(mg)	15	272.7	125.5	18.18	8.37	0(0,0)
Zinc(mg)	7.2	9.7	6.0	1.35	0.84	28.8(24.7,32.8)
Selenium (mcg)	42	65.7	42.9	1.56	1.02	13.7(10.7,17.0)
MAR (<75%)		67.7				72.3(67.9,76.5)

RDA, Recommended Daily Allowance; NAR, Nutrient Adequacy Ratio; MAR, Mean Adequacy Ratio; IQR, Interquartile Range; Q, Quartile; CI, Confidence Interval.

### Factors associated with micronutrient intake inadequacy

Age of the mothers, educational status of the mothers, occupation of the partner, household food security, and nutrition-related attitude were significantly associated with micronutrient intake inadequacy (MAR <0.75). The odds of micronutrient intake inadequacy were 2.5 times higher among lactating mothers aged 18–25 years old as compared to mothers in the age group ≥36 years old (AOR = 2.52, 95% CI: 1.09, 5.83). Mothers with the educational status of unable to read and primary school incomplete were 3.5 (AOR = 3.49, 95% CI: 1.24, 9.83) and 3.6 (AOR = 3.56, 95% CI: 1.06, 11.99) times more likely to have micronutrient intake inadequacy as compared to lactating mothers who had completed secondary school or above, respectively. Lactating mothers whose partners occupation was other than farming were 3.3 times more likely to have micronutrient intake inadequacy compared to mothers whose partners were farmers (AOR = 3.32, 95% CI: 1.08, 10.27). Lactating mothers who were from food-insecure households were 83% more likely to have high micronutrient intake inadequacy as compared to lactating mothers from food-secure households (AOR = 1.83, 95% CI: 1.04, 3.23). Lactating mothers with nutrition-related unfavorable attitudes were 77% more likely to have inadequate intake of micronutrients compared to lactating mothers with favorable attitudes (AOR = 1.77, 95% CI: 1.07, 2.93) (Table 4).

**Table 4:** Factors associated with micronutrient intake inadequacy among lactating mothers in North Mecha District, Amhara Region, Ethiopia, 2023 (*N* = 430).

Variables		Inadequate *n* (%)	Adequate *n* (%)	COR (95% CI)	AOR (95% CI)
Maternal age in years					
	18–25	94(79.0)	25(21.0)	2.10(1.03,4.29)*	2.52(1.09,5.83)*
	26–35	183(70.9)	75(29.1)	1.36(0.73,2.54)	1.41(0.73,2.71)
	≥36	34(64.2)	19(35.8)	1	1
Maternal education					
	Unable to read and write	233(73.0)	86(27.0)	1.91(0.88,4.17)	3.49(1.24,9.83)*
	Primary school incomplete	31(77.5)	9(22.5)	2.43(0.85,6.93)	3.56(1.06,11.99)*
	Primary school completed	30(71.4)	12(28.6)	1.77(0.65,4.78)	2.22(0.71,6.97)
	Secondary school completed or above	17(58.6)	12(41.4)	1	1
Partner occupation (423)					
	Farmer	278(71.3)	112(28.7)	1	1
	Other^#^	28(84.8)	5(15.2)	2.26(0.85,5.99)	3.32(1.08,10.27)*
Wealth Index					
	Poorest	62(72.1)	24(27.9)	1	
	Poor	55(65.5)	29(34.5)	0.73(0.38,1.41)	0.73(0.33,1.59)
	Medium	57(67.1)	28(32.9)	0.79(0.41,1.51)	0.81(0.38,1.76)
	Rich	66(73.3)	24(26.7)	1.07(0.55,2.07)	1.08(0.49,2.35)
	Richest	71(83.5)	14(16.5)	1.96(0.94,4.12)	1.66(0.71,3.86)
Household food security					
	Food Secure	223(69.5)	98(30.5)	1	1
	Food Insecure	88(80.7)	21(19.3)	1.84(1.08,3.14)*	1.83(1.04,3.23)*
Nutrition-related knowledge					
	Adequate	48(63.2)	28(36.8)	1	1
	Inadequate	263(74.3)	91(25.7)	1.69(0.99,2.85)	1.40(0.78,2.51)
Nutrition-related attitude					
	Favorable	199(69.1)	89(30.9)	1	1
	Unfavorable	112(78.9)	30(21.1)	1.67(1.04,2.68)*	1.77(1.07,2.93)*

*p-value < 0.05, 1: reference group, COR, Crude Odds Ratio; AOR, Adjusted Odds Ratio; CI, Confidence Interval. ^#^Merchant, Student, Daily laborer, Driver, Soldier, Veterinarian.

## Discussion

Micronutrient intake inadequacy among lactating mothers is an attribute of maternal and child malnutrition. This study addressed the paucity of evidence on micronutrient inadequacy among lactating mothers in rural Ethiopia, highlighting a significant public health concern. This study aimed to determine micronutrient intake inadequacy and its associated factors among lactating mothers in North Mecha District, Northwest Ethiopia.

It was observed that the MAR was between 0.29 and 0.94, and none of the lactating mothers met the RDA for all the micronutrients except for iron, which is consistent with the reports of other studies in Ethiopia and Zambia (11, 14). This study also revealed that the overall prevalence of micronutrient intake inadequacy (MAR <0.75) among lactating mothers was 72.3%. This is much higher than the findings of the study, which were in Bahir Dar City, Ethiopia (14) and in rural areas of Indonesia (41). This high prevalence of micronutrient intake inadequacy can be explained by factors such as excessive cereal consumption (teff, maize, sorghum, etc.), which has a low micronutrient density except for iron, which is abundant in these staples, and insufficient consumption of food items from other food groups such as animal-source foods, pulses, fruits, vegetables, and nuts and seeds, which are high in important micronutrients (42).

As compared to a study in Ethiopia (14), the difference may be explained by the differences in the study settings. Participants in this study reside in rural areas, whereas the previous study done in Ethiopia was conducted in the city of Bahir Dar, where people are more educated and therefore more aware of how to improve their dietary practices. In contrast to a study conducted in Indonesia (41), which computed MAR considering EAR (Estimated Average Requirement), which represents approximately 50% of the healthy individuals, the current study computed MAR considering RDA, which represents the minimum nutrient requirement of approximately 97.5% of the healthy individuals in a given life stage and gender group. When RDA is used instead of EAR, a higher level of nutrients must be consumed; this raises the prevalence of inadequacy due to the increased difficulty of meeting the RDA (25). The difference between using RDA or EAR is apparent in the calculation of NAR and determining the prevalence of nutrient inadequacy for each specific nutrient. Other researchers have reported disparities between studies using RDA (14, 43, 44) and EAR (8, 10–12, 41, 45).

Vitamin A intake inadequacy was prevalent among 86.7% of lactating mothers, according to this study. This is comparable to studies conducted in Ethiopia (13), Niger (12), and Bangladesh (8). In contrast, this study finding is lower than studies conducted in Bahir Dar City, Ethiopia (14), Zambia (11), China (45), and California (10), and higher than reports from studies conducted in Nigeria (46), Indonesia (41), Thailand (44) and Iran (43). Variations in the production and consumption practices of vitamin A-rich food sources, such as green leafy vegetables, could be a possible explanation for the potential difference (23, 47).

This study revealed that the prevalence of micronutrient intake inadequacy among selected B vitamins (vitamin B1, vitamin B2, vitamin B3, vitamin B6, vitamin B9, and vitamin B12) ranges from 18.1 to 82.3%. Among lactating mothers, 33.7% of them had inadequate intake for vitamin B1 (Thiamin), which is higher than studies in Ethiopia (13, 14), Iran (43), and California (10), and lower than findings from Zambia (11), Niger (12), Bangladesh (8), Thailand (44), China (45), and Indonesia (41). Dietary practices and mandatory fortification of staple foods have a major impact on thiamin intake; areas high in meat, whole grains, and legume production and consumption experience lower rates of inadequacy (48). Ethiopia has a low meat consumption rate, and the country lacks similar thiamin fortification programs (49).

This study showed that vitamin B2 (Riboflavin) intake inadequacy was found in 57.4% of participants. According to other studies conducted, this finding is consistent with a study in Ethiopia (14), lower than studies in Zambia (11) and Niger (12), Bangladesh (8), China (45), and higher than studies in Thailand (44), Indonesia (41), Iran (43), and California (43). Different countries have diverse dietary habits and riboflavin-rich foods available, such as organ and lean meats, dairy products, eggs, fortified cereals, and leafy green vegetables, leading to differences in inadequacy rates. Ethiopia, in particular, faces challenges in terms of poor consumption of foods rich in riboflavin.

The study revealed that 80.5% of mothers had insufficient amounts of vitamin B3 (Niacin). This is less than the results of a study conducted in Niger (12) but greater than reports from Ethiopia (13, 14), Zambia (11), Bangladesh (8), China (45), Indonesia (41), Iran (43), and California (10). In comparison to almost all the above study findings, the prevalence of niacin intake inadequacy is higher in rural lactating mothers. In Ethiopia, consumption of foods that are rich in niacin or tryptophan, a precursor to niacin synthesis, is modest (13, 14). Furthermore, maize-based foods account for a substantial amount of dishes in the study area, and maize is low in niacin and tryptophan.

The study found that 18.1% of participants lacked adequate vitamin B6 (Pyridoxine); less than study results in Ethiopia (13), California (10), and higher percentages were found in Ethiopia (14), Zambia (11), Niger (12), Thailand (44), China (45), Indonesia (41) and Iran (43). Ethiopian cuisine traditionally includes whole grains and legumes, as well as fermented foods like injera (50). Though not as rich as animal food sources, these food sources are good sources of pyridoxine, which could contribute to the relatively low pyridoxine intake inadequacy as compared to other studies.

Vitamin B9 (Folate) inadequacy was prevalent among 60.7% of the current study participants. The result of this study was similar to study findings in Iran (43), lower than reports in Zambia (11), Niger (12), Bangladesh (8), China (45), and California (10), and higher than findings in Ethiopia (14) and Indonesia (41). Though consumption of Ethiopian kale increases during certain months, consumption of dark green leafy vegetables and fruits is generally low in many other parts of Ethiopia as well as in the study area (51). While access to wild fruits is decreasing nowadays, even those that are available are underutilized (52). The possible difference across the findings of the studies could be due to variations in production and seasonal variations. There is limited availability of certain foods during specific seasons, such as vegetables in the winter and autumn.

This study revealed that vitamin B12 (Cobalamin) inadequacy was 82.3%. It is lower than studies in Ethiopia (13) and Niger (12), and higher than findings in Ethiopia (14), Zambia (11), Thailand (44), China (45), Thailand (41), Iran (43) and, California (10). Vitamin B12 is indeed mainly found in animal food sources, such as meat, fish, poultry, eggs, and dairy products. In rural Ethiopia, where the diet predominantly consists of plant-based foods, this limited access to animal food sources would result in an increase in vitamin B12 inadequacy. In general, along with Vitamin B12, potential differences in the inadequacy of the above-mentioned B vitamins among studies conducted in different countries could be attributed to differences in dietary patterns, food production and availability, education levels and awareness, and socioeconomic status to access diverse food options.

When compared to all other micronutrients, nearly all mothers (98.4%) undoubtedly failed to meet the RDA for vitamin C. The finding corresponded to the Niger result (12); lower reports were obtained in Ethiopia (13, 14), Zambia (11), Bangladesh (8), Thailand (44), China (45), Indonesia (41), Iran (43), and California (10). This could be explained by the variation in fruit and vegetable consumption, as the current study showed quite low fruit and vegetable consumption.

About 92.8% of study subjects were inadequate in calcium intake. The result was similar to a study result in Bangladesh (8), lower than study results in Ethiopia (13), Zambia (11) and Niger (12), and higher than Ethiopia (14), Thailand (44), China (45), Indonesia (41), Iran (43) and California (10). The observed difference may be due to variations in consumption of dairy products and other calcium-rich foods, whose consumption was low in this study area.

Despite the high prevalence of iron deficiency anemia among mothers in Ethiopia (17, 53), surprisingly, this study showed that all mothers highly exceeded the requirements for iron, and none of the study subjects were found to be inadequate for iron. This is consistent with the findings of other studies conducted in Ethiopia (14), Iran (43), and California (10). However, inadequate intake was also reported in other studies in Ethiopia (13), Zambia (11), Niger (12), Bangladesh (8), Thailand (44), China (45), and Indonesia (41). The difference may be due to the high consumption of cereals, grains, and legumes in the study area, which are the major sources of iron even in the presence of antinutrients. In the study area, teff and millet were staple foods that are rich sources of iron. Despite this iron adequateness, the higher content of antinutrients and fiber in plant-based diets results in the low bioavailability of iron and possibly certain nutrients. This potentially explains the high incidence of anemia (21).

According to this study, zinc intake inadequacy was 28.8%, which is lower than studies in Ethiopia (13), Zambia (11), Bangladesh (8), Thailand (44), China (45), Iran (43), and higher than Ethiopia (14), Niger (12), Indonesia (41) and California (10). This discrepancy could be attributed to variations in the kinds of foods eaten in various regions, where zinc concentrations vary correspondingly.

Selenium intake inadequacy was prevalent among 13.7% of lactating mothers in the present study. This prevalence is greater than study findings in California (10) and lower than reports in Ethiopia (14) and Thailand (44). Different regions have distinct dietary patterns and selenium sources, leading to variations in intake levels. In addition, selenium content in soil and crops varies depending on geographical location (54).

In general, with regard to differences in study results of micronutrient intake in lactating mothers, numerous factors would contribute to the variability. These include differences in dietary intake measures, assessment methods, and indicator score cutoffs; population characteristics explained with variations in demographic characteristics, cultural practices, and socioeconomic status; geographical variation in types of food production and soil concentration of the nutrients (14, 25, 27, 28, 54, 55).

The odds of micronutrient intake inadequacy were 2.5 times higher among lactating mothers aged 18–25 years old as compared to mothers in the age group ≥36 years old. Younger lactating mothers potentially face financial constraints or have a lower socioeconomic status when compared to older mothers (56). Consequently, they may encounter challenges in accessing nutrient-dense foods. Furthermore, nutrition-related knowledge and attitudes are crucial factors, with older mothers having potentially greater exposure to nutrition-related information, empowering them to make informed decisions regarding their dietary choices.

Mothers with educational status of unable to read and write and primary school incomplete were 3.5 and 3.6 times more likely to have micronutrient intake inadequacy than mothers with secondary school completed or above educational status, respectively, as supported by other studies in Indonesia and Bangladesh (8, 41). Education empowers mothers to make informed decisions about diet and health, improving their understanding, attitude, healthcare inclination, and adherence to nutrition education (56–58).

Mothers whose partners had occupations other than farming were 3.3 times more likely to experience inadequate intake of essential micronutrients, when compared to mothers whose partners were farmers. This disparity can be attributed to the fact that farmers have better opportunities to cultivate diverse crops and access a wide range of fruits and vegetables, while those without farming occupations rely on market purchases.

The likelihood of micronutrient inadequacy was 1.8 times higher among mothers from food-insecure households than their counterparts. Food scarcity in households experiencing food insecurity reduces the likelihood of accessing a diverse range of nutritious foods (24, 58–60). As a result, lactating mothers face an increased risk of nutrient deficiency.

Mothers who held an unfavorable attitude toward nutrition were found to be 1.8 times more likely to have inadequate intake of essential micronutrients compared to those with a favorable attitude. A woman with a favorable nutrition-related attitude could have a high chance of adhering to nutrition education and striding to fulfill the recommended nutrient requirements accordingly, which would motivate her to consume a diverse and balanced diet (33).

This community-based study on the inadequacy of micronutrient intake provided an impressive picture of the nutrient intake of lactating mothers, whose consumption dictates the nutritional status of the mother–child pair, which could have a negative impact on a mother’s present health and future susceptibility to disease, as well as a child’s growth, development, and overall health, including later in life. However, relying solely on a single 24-h recall may lead to potential inaccuracies due to variations in day-to-day dietary patterns and memory biases. To mitigate this bias, maximum efforts were exerted by incorporating standardized quality-control procedures using well-trained nutrition experts throughout the entire study process and not counting days of special events. Moreover, the method is validated for low- and middle-income countries (61). There were certain challenges faced concerning food composition tables, including limited accessibility to specific food items that account for diverse cultural preferences as well as the absence of certain essential nutrients in the tables. To overcome these challenges, besides the exhaustive efforts made to identify closely resembling food items, recipes were prepared to replicate the desired food items. Additionally, apart from relying on the Ethiopian food composition tables, references were also made to tables from different countries (36–39).

## Conclusion

Seven out of 10 lactating mothers in rural kebeles in the Amhara Region had inadequate micronutrient intake. With the exception of iron, all micronutrient intakes by lactating mothers did not meet the recommended levels. Age of the mothers, educational status of the mothers, occupation of the partner, household food security, and nutrition-related attitude were significantly associated with micronutrient intake inadequacy. Community driven nutrition education and interventions are needed to address the high micronutrient intake inadequacy among lactating mothers in rural Ethiopia. As a result, it is possible to raise awareness about their heightened nutrient requirements, improve their attitudes toward nutrition, and empower them to make informed dietary decisions.

## Data availability statement

The datasets presented in this article are not readily available because the data set is needed for further analysis. However, if a reasonable request is made, the data set containing specific variables will be made available. Requests to access the datasets should be directed to YM, nataniem21@gmail.com.

## Ethics statement

The study involving humans was approved by the Ethical Review Board of the College of Medicine and Health Sciences at Bahir Dar University. The studies were conducted in accordance with the local legislation and institutional requirements. The participants provided their written informed consent to participate in this study.

## Author contributions

YM: Conceptualization, Data curation, Formal analysis, Investigation, Methodology, Project administration, Software, Supervision, Validation, Visualization, Writing – original draft, Writing – review & editing. SG: Conceptualization, Data curation, Formal analysis, Investigation, Methodology, Software, Supervision, Validation, Visualization, Writing – original draft. TB: Conceptualization, Investigation, Methodology, Supervision, Validation, Visualization, Writing – review & editing. NF: Conceptualization, Investigation, Methodology, Project administration, Supervision, Validation, Visualization, Writing – review & editing.
